# The Clinical Significance of Serum Apoptotic Cytokeratin 18 Neoepitope M30 (CK-18 M30) and Matrix Metalloproteinase 2 (MMP-2) Levels in Chronic Hepatitis B Patients with Cirrhosis

**DOI:** 10.5812/hepatmon.10106

**Published:** 2013-06-26

**Authors:** Sua Sumer, Nazlim Aktug Demir, Servet Kölgelier, Ahmet Cagkan Inkaya, Abdullah Arpaci, Lütfi Saltuk Demir, Onur Ural

**Affiliations:** 1 Department of Infectious Diseases and Clinical Microbiology, Selcuk University, Faculty of Medicine, Konya, Turkey; 2 Department of Infectious Diseases and Clinical Microbiology, Adiyaman State Hospital, Adiyaman, Turkey; 3 Department of Infectious Diseases and Clinical Microbiology, Adiyaman University, Faculty of Medicine, Adiyaman, Turkey; 4 Department of Internal Medicine, Division of Infectious Diseases, Hacettepe University, Faculty of Medicine, Ankara, Turkey; 5 Department of Biochemistry, Adiyaman University, Faculty of Medicine, Adiyaman, Turkey; 6 Public Health Management Center, Konya, Turkey

**Keywords:** Hepatitis B, Chronic, Cirrhosis, Liver Cirrhosis, Matrix Metalloproteinase 2

## Abstract

**Background:**

Serum apoptotic cytokeratine 18 neoepitope M30 (CK-18 M30) and matrix metalloproteinase 2 (MMP-2) have been popular markers for detecting liver fibrosis in recent years. CK-18 is a major intermediate filament protein in liver cells and one of the most prominent substrates of caspases during hepatocyte apoptosis. MMP-2 plays an important role in tissue remodeling and repairing processes during physiological and pathological states.

**Objectives:**

The objective of this study was to investigate the significance of CK-18 M30 and MMP-2 levels for clinical use in patients with chronic hepatitis B (CHB), as well as their sensitivity in determining cirrhotic patients.

**Patients and Methods:**

This study included 189 CHB patients and 51 healthy controls. A modified Knodell scoring system was used to determine the fibrosis level in chronic hepatitis B patients. CK-18 M30 levels were determined with an M30-Apoptosense ELISA assay. MMP-2 levels were determined with the ELISA assay.

**Results:**

The study group consisted of 132 (69.8%) males and 57 (30.2%) females, and the control group consisted of 25 males (49.0%) and 26 females (51%). Patients’ CK-18 M30 levels were higher than values of the control group (308 [1–762] vs. 168 [67–287], P=0.001). Serum MMP-2 levels were found to be statistically higher in the patient group with respect to the controls (3.0 [1.1–6.8] vs. 2.0 [1.2–3.4], P=0.001). The highest serum CK-18 M30 and MMP-2 levels were measured in patients with cirrhosis. Serum apoptotic CK-18 M30 levels positively correlated with advanced age, fibrosis stage, serum alanine aminotransferase (ALT) and aspartate aminotransferase (AST) levels (P= 0.001, 0.033, 0.001, and 0.001, respectively). Serum MMP-2 levels positively correlated with fibrosis stage, serum ALT, and AST levels (P= 0.001, 0.001, and 0.001, respectively).

**Conclusions:**

Our study indicated that CK-18 M30 and MMP-2 levels were higher in CHB patients compared to healthy controls and they were in association with significant hepatic fibrosis, especially cirrhosis.

## 1. Background

Chronic hepatitis B (CHB) infection, which represents one of the most common causes of chronic liver disease worldwide, is associated with increased risk for cirrhosis and hepatocellular carcinoma (1-3). In CHB infection, liver histology may be normal, mild or moderately damaged, or cirrhotic. A serious increase in liver fibrosis is observed in the advanced stages of the disease (4). Liver biopsy, which is an invasive method used to determine fibrosis in the liver, may lead to complications (5-9). Therefore, noninvasive markers have recently come into prominence to determine the level of fibrosis. Serum apoptotic cytokeratine 18 neoepitope M30 (CK-18 M30) and matrix metalloproteinase 2 (MMP-2) are among the popular noninvasive markers used to detect liver fibrosis. Hepatocyte apoptosis can be initiated by different complex pathways, most of which involve activation of the caspases(10, 11). CK-18 is a major intermediate filament protein in liver cells and one of the most prominent substrates of caspases during hepatocyte apoptosis (4, 10, 12). CK-18 M30, which is coded between the 387, 396th position of CK-18 is produced by caspase system activation and accepted as an apoptosis marker (10, 13, 14). Altered degradation and increased synthesis of extracellular matrix (ECM) proteins are implicated in liver fibrosis (15-17). Matrix metalloproteinases (MMPs) are the main degrading enzymes of ECM proteins and play important roles in the tissue remodeling and repairing processes during the physiological and pathological states (17, 18). MMP-2 decreases natural collagen types and denatures interstitial collagens. Here, the level of type IV collagen decreases; type IV collagen has an important role in basal membrane remodeling and turnover (17, 19).The M30 fragment of CK-18 has been identified as a useful marker associated with increased severity of fibrosis and inflammation in chronic hepatitis C (CHC) and nonalcoholic fatty liver disease (NAFLD) (20). Research on MMP-2 is on-going. The diagnostic importance of these markers for CHB infection is still not clear.

## 2. Objectives

The objective of this study is to investigate the significance of CK-18 M30 and MMP-2 levels in clinical use in patients with chronic hepatitis B (CHB), as well as their sensitivity in determining cirrhotic patients.

## 3. Patients and Methods

### 3.1. Patients

This study included 189 CHB patients (HBeAg-negative) and 51 healthy controls admitted to infectious diseases clinics of Adiyaman State Hospital, Adiyaman 82nd Year State Hospital, and Adana State Hospital between January 1st and December 31st, 2010. Demographic features of patients were recorded on follow-up sheets. The control group included healthy volunteers whose alanine aminotransferase (ALT) and aspartate aminotransferase (AST) values were normal, and who were found to be negative for HBsAg and anti-HCV.

### 3.2. Preparation for Liver Biopsy

Pre-biopsy hepatitis markers, ALT, AST, HBV DNA levels, platelet counts, and PT levels of all patients were investigated. Liver ultrasonography was performed. Patients with PT ≥ 1.5 INR and platelet count ≤ 50.000/mm3 were excluded from the study.

### 3.3. Liver Biopsy

Biopsy criteria were established according to American Association for the Study of Liver Diseases (AASLD) criteria. Liver biopsies that were sent to the pathology laboratory within a formaldehyde fixation solution with a chronic hepatitis B prediagnosis were examined. All samples were needle biopsy material and their lengths were 1–3 cm. Sections from the samples, which were stained by hematoxylin-eosin, reticulin, and Masson trichrome, were examined under light microscopy by two pathologists using the Ishak modified histologic activity index. Biopsies of CHB patients were reported in terms of fibrosis score.

### 3.4. Determination of CK-18 M30 and MMP-2

After liver biopsy, a 5 cc blood sample was taken from each patient in the same day. Serum was separated via centrifugation at 5,000 cycles/minute for 3 minutes. Serum samples were kept at -80°C. CK-18 M30 levels were determined through M30-Apoptosense ELISA assay (PEVIVA, Alexis, Grünwald, Germany) from serum. MMP-2 levels were determined using the quantitative sandwich enzyme immunoassay technique (Quantikine Human MMP-2 Immunoassay, R&D Systems, Minneapolis, USA) from serum.

### 3.5. Virological and Biochemical Parameters

Markers for HBsAg, anti-HBs, HBeAg, and anti-Hbe were measured by Makro ELISA (Abbott AXSYM SYSTEM, Germany). HBV DNA testing was conducted using the real-time PCR (ICycler IQ Real-Time PCR; BioRad, USA) method. Biochemical parameters such as ALT, AST, and albumin levels were measured using an Abbott Architect plus c16000 device. Sysmex XT 2000i (Roche) was used to measure hemograms. Alpha-fetoprotein (AFP) levels were measured using a Modular E170 (Roche) device.

### 3.6. Ethics

This work was carried out in accordance with the Declaration of Helsinki (2000) of the World Medical Association. Approval was obtained from the ethical committee of Adiyaman University (2012/02-4.4). Informed consent was received from all patients involved in this study.

### 3.7. Statistical analyses

Data on the study participants were analyzed using the SPSS 18.0 software. Descriptive statistics included mean ± standard deviation, median (minimum and maximum values), and percentage distribution. The independent samples T test and Mann-Whitney U test were used for statistical analyses. To analyze the association of fibrosis stages with CK-18 M30 and MMP-2, the Mann-Whitney U test with Bonferroni correction was used after the Kruskal-Wallis test. Pearson and Spearman correlations were used to determine the correlations between different variables. A value of P < 0.05 was considered statistically significant.

## 4. Results

**Figure 1.**
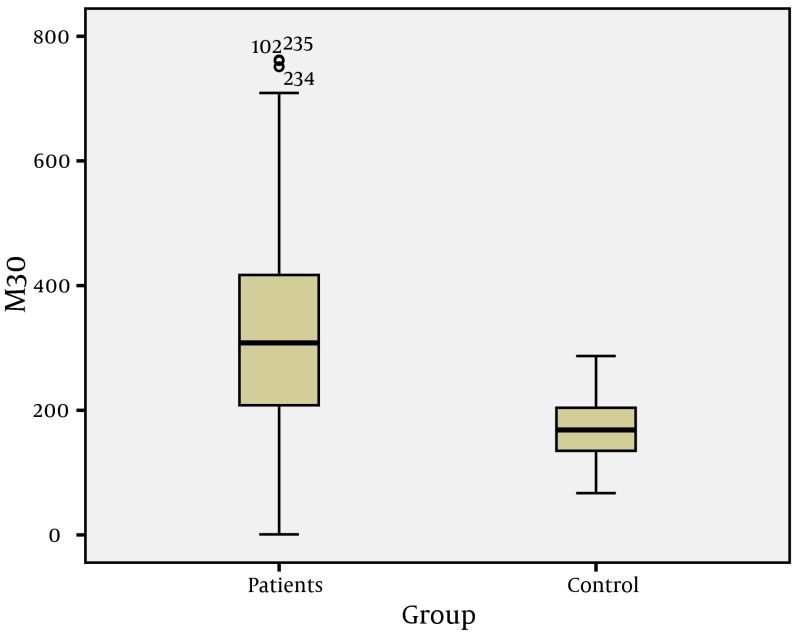
CK-18 M30 Values of Patients and Control Groups

**Figure 2.**
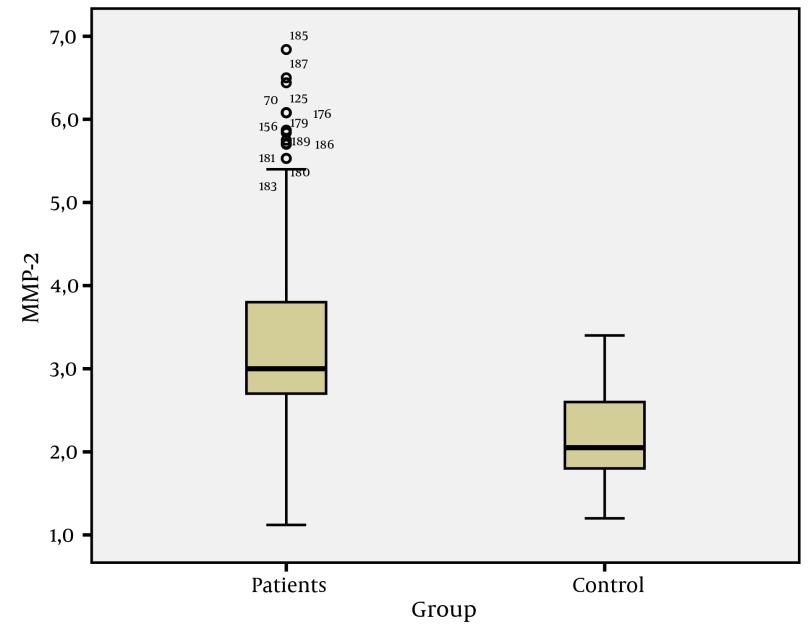
MMP-2 Values of Patients and Control Groups

The study group consisted of 132 (69.8%) males and 57 (30.2%) females, and the control group consisted of 25 males (49.0%) and 26 females (51%) (P > 0.05). Mean age of patients was 37.8 ± 12.6 andfor controls, this was 33.8 ± 9.3 (P > 0.05) (Table 1). In general, the CK-18 M30 and MMP-2 values of patients were higher than values of the control group (P = 0.001) (Figure 1 and 2).

**Table 1.** CK-18 M30 and MMP-2 Values of Patients and Control Groups

	Patient Group (n = 189)	Control Group (n = 51)	P value
**CK-18 M30 (U/L) ^a^**	308 (1-762)	168 (67-287)	0.001
**MMP-2 (ng/ml) ^a^**	3.0 (1.1-6.8)	2.0 (1.2-3.4)	0.001

^a^CK-18, is a major intermediate filament protein in liver cells and one of the most prominent substrates of caspases during hepatocyte apoptosis; MMP-2, repairing processes during the physiological and pathological states

The CK-18 M30 and MMP-2 values of patients whose liver biopsy revealed a fibrosis stage of 1, 2, 3, 4, or 5 were higher than the CK-18 M30 and MMP-2 values of controls ( P = 0.01, P = 0.01, P = 0.01, P = 0.001, and P = 0.01, respectively). The associations of fibrosis stages with HBV DNA, AST, ALT, and platelet levels were statistically significant (P < 0.001) (Table 2).

**Table 2.** CK-18 M30, MMP-2, ALT, AST, HBV DNA, AFP, PLT, and Albumin Levels Versus Fibrosis Stages at Liver Biopsy

	Stage 1, (n = 40)	Stage 2, (n = 77)	Stage 3, (n = 37)	Stage 4, (n = 230	Stage 5, (n = 12)	P value
**CK-18 M30 (U/L)**	168 (1-292)	259 (102-761)	396 (259-598)	462 (400-598)	689 (559-762)	0.001
**MMP-2 (ng/ml)**	2.7 (1.1-6.1)	2.8 (2.0-6.1)	3.4 (2.2-6.1)	4.6 (2.6-6.1)	5.7 (2.4-6.8)	0.001
**ALT ^a^**	77.5 (16-476)	90 (14-665)	124.5 (25-476)	120 (37-346)	122 (25-219)	0.001
**AST ^a^**	50.5 (15-230)	77 (20-552)	102 (35-322)	96 (35-350)	112 (21-205)	0.001
**HBV ^a^ DNA**	4.5x10^7^(6x10^4^-7.7x10^9^)	9.3x10^7^ (2.4x10^5^-3.4x10^10^)	1.1x10^9^ (1.1x10^5^-3.3x10^9^)	3.8x10^7^ (3.9x10^6^-9x10^9^)	2.8x10^7^ (1x10^6^-7.2x10^8^)	0.001
**AFP ^a^**	2.8 (1-14)	3.1 (1-12)	3.3(1-9)	2.9 (1-5)	3.1(1-4)	0.110
**Platelet**	2.7x10^5^ (1.1x10^5^-4.1x10^5^)	2.5x10^5^ (1.4x10^5^- 4.2x10^5^)	2x10^5^ (8.9x10^4^-4.5x10^5^)	1.8x10^5^ (7x10^4^-3.5x10^5^)	1.9x10^5^ (8.8x10^4^-4x10^5^)	0.001
**Albumin**	3.8 (3.2-5.0)	3.9 (3.3-5.0)	3.8 (3.4-4.9)	3.9 (3.0-4.3)	3.7 (3.0-4.6)	0.483

^a^Abbreviations: AFP, alpha-fetoprotein; ALT, serum alanine aminotransferase; AST, aspartate aminotransferase; HBV, hepatitis B virus

**Figure 3.**
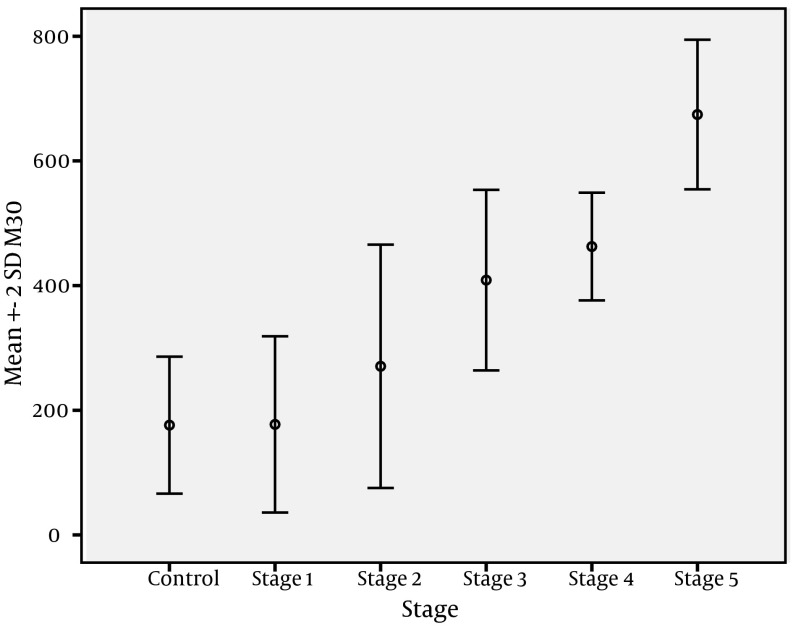
Change in CK-18 M30 Levels According to Fibrosis Stages

**Figure 4.**
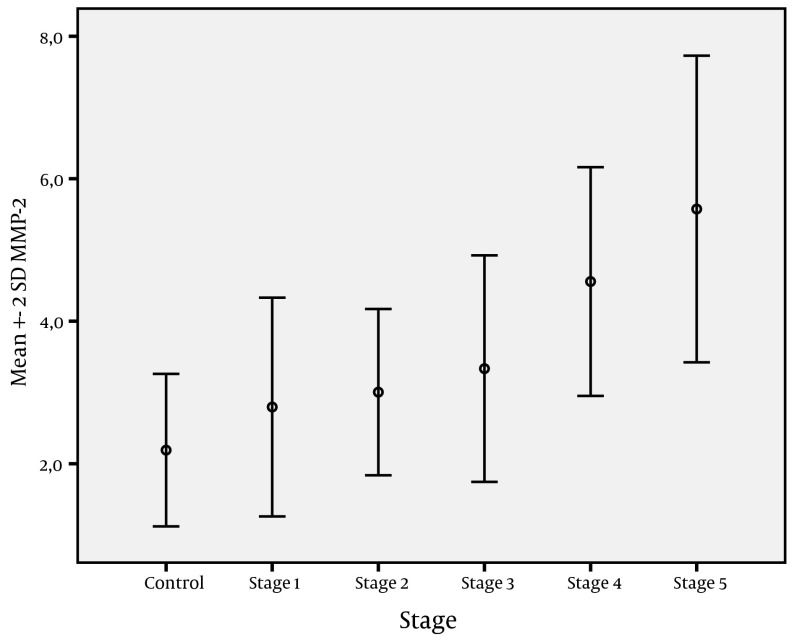
Change in MMP-2 Levels According to Fibrosis Stages

Figure 3 shows the change in CK-18 M30 levels with fibrosis stages, while Figure 4 shows change of MMP-2 levels with fibrosis stages. The change in CK-18 M30 level with fibrosis stage was significant for each stage (P = 0.001). Evaluation of change in MMP-2 levels according to fibrosis stage showed that no significant difference was present between stage 1 and 2 or stage 2 and 3, but there was a significant difference between stage 4 and 5 (P=0.001). CK-18 M30 was positively correlated with age, stage, ALT, and AST levels (P values were 0.033, 0.001, 0.001, and 0.004, respectively). On the other hand, MMP-2 level was positively correlated with stage, ALT, and AST (P values were 0.001, 0.001, and 0.012, respectively).

## 5. Discussion

CK-18 M30 and MMP-2 are very interesting markers for chronic hepatitis. Previous studies on CK-18 M30 and MMP-2 have generally focused on CHC and NAFLD.

In these studies, CK-18 M30 and MMP-2 levels were reported to be sensitive markers that can be used to determine the level of fibrosis ( 12 , 20 , 23 ). However, only a few studies have evaluated CK-18 M30 and MMP-2 levels in CHB patients ( 22 , 24 , 25 ).Two important features should be considered in noninvasive markers used to determine the fibrosis levels of chronic viral hepatitis. The first is that they must detect fibrosis accurately, and the second is that they must reflect changes in fibrosis level according to fibrosis stage. In our study, statistically significant differences in CK-18 M30 and MMP-2 levels between CHB patients and the control group (P = 0.001) indicated that both markers can be used to show liver damage (Figure 1 and 2). Similar to our findings, Jazwinski et al. ( 26 ) reported that CK-18 levels were significantly higher in CHC patients than in controls. Kronenberger et al. ( 27 ) found that CK-18 levels in patients with CHC and persistently normal ALT levels were significantly higher than in controls. Ljumovic et al. ( 10 ) found that MMP-2 levels were increased in 66% of chronic hepatitis patients as a result of viral or nonviral causes compared to healthy controls. On the other hand, Murawaki et al. ( 17 ) found no difference in MMP-2 levels between chronic hepatitis B group and healthy controls. In our study, the CK-18 M30 levels of patients with CHB infection were different at each stage. However, evaluation of MMP-2 levels showed no difference between stage 1 and 2 and stage 2 and 3; on the other hand, there were statistically significant differences between the other groups. This study showed that CK-18 M30 is a more sensitive marker than MMP-2 for predicting the histological stage of fibrosis. Shi et al. ( 28 ) reported that creatine 18 phosphorylation is a progression marker in CHB. On the other hand, Papatheodoridis et al. ( 25 ) reported that the change in the serum CK-18 fragment level was not sufficient to determine the severity of histological lesions.

In a MMP-2 study, Liang et al. ( 29 ) were unable to find a significant difference between CHB patients at early fibrosis stages and MMP-2 levels, but did observe a significant difference at advanced stages. Similarly, Murawaki et al. ( 17 ) found no difference in MMP-2 levels according to the chronic hepatitis stages.HBV DNA, AST, ALT, AFP, platelet, and albumin levels are still useful markers for follow-up of CHB. However, it is not possible to determine the level of liver fibrosis by assessing these markers alone. Upon evaluation, HBV DNA, AST, ALT, AFP, platelet, and albumin levels were found to be statistically significant according to the level of fibrosis (Table 2). Additionally, CK-18 M30 and MMP-2 levels were positively correlated with ALT and AST levels. There was a positive correlation only between CK-18 M30 and age. Similarly, Bantel et al. ( 20 ) and Kronenberger et al. ( 27 ) found strong correlations in CHC patients between CK-18 and ALT levels. Cirrhosis is the stage where apoptosis and therefore fibrosis is seen in its most severe form in patients with chronic fibrosis ( 30 ). This causes increased levels of CK-18 M30 and MMP-2. Research on this topic supports this claim. Kronenberger et al. ( 27 ) detected the highest serum CK-18 levels in cirrhotic CHC patients, while Murawaki et al. ( 17 ) reported that in patients with chronic viral liver disease, the serum MMP-2 level increases in the presence of cirrhosis and HCC. Thus, MMP-2 is a useful diagnostic test, especially when it comes to detecting cirrhosis. Boeker et al. ( 23 ) evaluated MMP-2 levels in CHC patients and found high sensitivity in cirrhotic patients. Our findings are concordant with these studies. According to our study findings, both CK-18 and MMP-2 levels increased in CHB patients and the most significant elevations were detected in cirrhotic patients (Figure 3 and 4).

Our study has some limitations. First, the correlation between the degree of inflammation in liver biopsy and CK-18 M30 and MMP-2 levels was not evaluated. Second, a cut-off value could not be defined for CK-18 M30 and MMP-2 levels.In conclusion, our study indicated that CK-18 M30 and MMP-2 levels are higher in CHB patients compared to healthy controls and are associated with significant hepatic fibrosis, especially cirrhosis. Although roles to detect liver fibrosis in CHB are still not known exactly, recent studies along with our own have reported promising results.
